# Systematic misclassification of missense variants in *BRCA1* and *BRCA2* “coldspots”

**DOI:** 10.1038/s41436-019-0740-6

**Published:** 2020-01-08

**Authors:** Jennifer N. Dines, Brian H. Shirts, Thomas P. Slavin, Tom Walsh, Mary-Claire King, Douglas M. Fowler, Colin C. Pritchard

**Affiliations:** 1 Department of Medicine, Division of Medical Genetics, University of Washington, Seattle, WA USA; 2 Department of Laboratory Medicine, University of Washington, Seattle, WA USA; 3 Department of Medical Oncology, Division of Clinical Cancer Genomics, City of Hope, Duarte, CA USA; 4 Department of Genome Sciences, University of Washington, Seattle, WA USA

**Keywords:** variant classification, VUS, coldspot, ACMG, *BRCA1*

## Abstract

**Purpose:**

Guidelines for variant interpretation incorporate variant hotspots in critical functional domains as evidence for pathogenicity (e.g., PM1 and PP2), but do not use “coldspots,” that is, regions without essential functions that tolerate variation, as evidence a variant is benign. To improve variant classification we evaluated *BRCA1* and *BRCA2* missense variants reported in ClinVar to identify regions where pathogenic missenses are extremely infrequent, defined as coldspots.

**Methods:**

We used Bayesian approaches to model variant classification in these regions.

**Results:**

*BRCA1* exon 11 (~60% of the coding sequence), and *BRCA2* exons 10 and 11 (~65% of the coding sequence), are coldspots. Of 89 pathogenic (P) or likely pathogenic (LP) missense variants in *BRCA1*, none are in exon 11 (odds <0.01, 95% confidence interval [CI] 0.0–0.01). Of 34 P or LP missense variants in *BRCA2*, none are in exons 10–11 (odds <0.01, 95% CI 0.0–0.01). More than half of reported missense variants of uncertain significance (VUS) in *BRCA1* and *BRCA2* are in coldspots (3115/5301 = 58.8%). Reclassifying these 3115 VUS as likely benign would substantially improve variant classification.

**Conclusion:**

In *BRCA1* and *BRCA2* coldspots, missense variants are very unlikely to be pathogenic. Classification schemes that incorporate coldspots can reduce the number of VUS and mitigate risks from reporting benign variation as VUS.

## INTRODUCTION

Panel genetic testing has led to an abundance of variants of uncertain significance (VUS), a designation for rare variation with insufficient evidence. Among 471,622 variants submitted to ClinVar, 221,846 are classified as VUS (accessed December 2018).^1^ VUS tend to be missense variants because the functional effect of missense variants is more challenging to deduce, as compared with frameshift or nonsense variants. Thus, rare missense variants are a particular challenge for interpretation, even in genes that are highly conserved.

Suboptimal outcomes can occur for individuals managed in the context of a VUS, particularly when medical providers do not have formal genetics training.^2^ A potent example is in *BRCA1/2*, where both surgical prevention and oncology treatment decisions are made based on germline pathogenic variants.^3–5^ National guidelines recommend against management decisions in the context of *BRCA1/2* VUS;^6,7^ however, in practice, VUS do drive changes in management.^2,8–10^ For this reason, there is an urgent need to improve variant classification to reduce the number of reported VUS.

We propose that when a missense variant occurs in a “coldspot,” a region of a gene that is more tolerant to variation, this provides evidence for classifying the variant as benign or likely benign as opposed to VUS. Coldspots correlate to an already established American College of Medical Genetics and Genomics/Association for Molecular Pathology (ACMG/AMP) criteria for classifying pathogenic variants (PM1: located in a variant hotspot and/or critical and well-established functional domain without benign variation^7^). Large regions of *BRCA1* and *BRCA2* are known to have a low probability of damaging missense variation;^11^ however, applying this knowledge to directly impact variant classification has not been done. Here, we analyzed 5720 missense variants in *BRCA1* and *BRCA2* and used a Bayesian approach to identify coldspots. Use of these coldspots suggests that over half of these VUS are more appropriately classified as likely benign. We propose that location within a coldspot be considered in variant classification guidelines as strong evidence that a missense variant in *BRCA1* and *BRCA2* is benign.

## MATERIALS AND METHODS

Missense variants from ClinVar^12^ in *BRCA1* (NM_007294.3) and *BRCA2* (NM_000059.3) were exported and curated for accuracy (queried October 2019) and placed into four categories according to their classifications: (1) P + LP (includes pathogenic and likely pathogenic variants; (2) B + LB (includes benign and likely benign variants); (3) VUS (variant of uncertain significance); and (4) CIP, variants with conflicting interpretation of pathogenicity (CIP) (Supplementary Data File). We defined “CIP Major” as a CIP that included at least one P/LP submission and at least one VUS/LB/B submission, and “CIP Minor” as CIP involving B/LB versus VUS. There were only four total CIP Major variants in coldspots, each classified as P/LP by a single submitter without evidence provided (Supplementary Data File). For the analysis, we excluded missense variants with CIP, no interpretation, no assertion criteria provided (0 star), or inaccurate annotation, as well as deletions and insertions affecting more than one codon, and start-loss variants. Only one interpretation was used per variant. For a complete list of variants used in the analysis see the Supplementary Data File. Unique variant counts were grouped in 10 amino acid increments and rolling averages were calculated as a percent of variants divided by the total number of variants per classification type in 50 amino acid increments (Eq. (1)).

1 $$\frac{{Rolling\,Average\,in\,50\,amino\,acid\,increments\left( {\frac{{\# \,of\,variants\,per\,classification\,type\,in\,10\,amino\,acid\,increments}}{{Total\,\# \,of\;variants\,per\,classification\,type}}} \right)}}{{SUM\,of\,Rolling\,averages\,per\,classification\,type}}$$

Critical functional domains were defined based on literature consensus for amino acid boundaries^13–15^ (Tables 1, 2). Exon 11 in *BRCA1* and exons 10 and 11 in *BRCA2* were considered potential coldspots, consistent with literature describing the lack of pathogenic missense variants outside of known critical domains.^11,16^

**Table 1:** ClinVar classification of missense variants in *BRCA1* and *BRCA2*.

Gene	Region	Codons	P or LP *N* (%)^a^	B or LB *N* (%)^a^	VUS *N* (%)^a^	Total *N* (%)^a^
*BRCA1*	Total missenses	1–1863	89 (4.5)	119 (6.0)	1759 (89.4)	1967 (100.0)
	RING domain	9–98	29 (24.8)	3 (2.6)	85 (72.6)	117 (100.0)
	BRCT repeats	1649–1859	53 (17.4)	19 (6.2)	233 (76.4)	305 (100.0)
	Exon 11	224–1366	0 (0)	69 (6.2)	1048 (93.8)	1117 (100.0)
	Coiled-coil	1393–1424	1 (2.4)	2 (4.9)	38 (92.7)	41 (100.0)
*BRCA2*	Total missenses	1–3418	34 (0.9)	177 (4.7)	3542 (94.4)	3753 (100.0)
	Exon 10 and 11	266–2281	0 (0)	110 (5.1)	2067 (94.9)	2177 (100.0)
	BRC repeats	1008–2082	0 (0)	53 (4.4)	1147 (95.6)	1200 (100.0)
	DNA binding	2481–3186	25 (3.0)	26 (3.1)	778 (93.8)	829 (100.0)

Transcripts are NM_007294.3 for *BRCA1* and NM_000059.3 for *BRCA2*. Variants were classified as described in the methods and last queried October 2019.

*B or LB* benign or likely benign, *P or LP* pathogenic or likely pathogenic, *VUS* variant of uncertain significance.

^a^Percent of P/LP, B/LB, and VUS in each region (by row), excluding variants with conflicting interpretations of pathogenicity.

**Table 2:** Odds ratios (OR) and 95% confidence intervals (CI) for pathogenicity of missense variants in *BRCA1* and *BRCA2*.

Gene	Region	Codons	OR (95% CI)^a^	Bayesian analysis
*BRCA1*	Ring domain	9–98	8.4 (6.4–11.2)	Supports pathogenic
	Exon 11	224–1366	<0.01 (0.0–0.01)	Strong benign (coldspot)
	Coiled-coil domain	1393–1424	0.6 (0.04–9.7)	Moderate benign
	BRCT repeats	1649–1859	2.7 (0.9–8.6)	Supports pathogenic
*BRCA2*	exon 10 and 11	266–2281	<0.01 (0.0–0.01)	Strong benign (coldspot)
	DNA-binding domain	2481–3186	1.0 (0.06–15.6)	Not informative

^a^Odds ratios are calculated as (P + LP + 1/2)/(B + LB + 1/2). For example, an odds ratio of 1.0 indicates the same number of P + LP and B + LB variants in a given region. Based on Bayesian analysis (Tavtigian et al.^18^), odds ratios <0.48 were considered "supporting benign," <0.23 "moderate benign,” and <0.05 "strong benign.”

Following the identification of potential coldspots, the odds of pathogenicity were tabulated for critical domains and coldspots separately from the ratio of percentage of total variants considered pathogenic (P + LP) to the percentage of total variants considered benign (B + LB). To avoid having a zero count in the numerator or denominator, we added +½ to each.

Odds (pathogenic) of a given region (critical domain or coldspot):

2 $$\frac{{\frac{{({\mathrm{P}} + {\mathrm{LP}}) + 1/2}}{{Total\,variants + 1/2}}}}{{\frac{{(1 - ({\mathrm{P}} + {\mathrm{LP}}) + 1/2)}}{{Total\,variants + 1/2}}}}$$

Odds (benign) of a given region (critical domain or coldspot):

3 $$\frac{{\frac{{({\mathrm{B}} + {\mathrm{LB}}) + 1/2}}{{Total\,variants + 1/2}}}}{{\frac{{(1 - ({\mathrm{B}} + {\mathrm{LB}}) + 1/2)}}{{Total\,variants + 1/2}}}}$$

Odds ratio of pathogenicity:

4 $$\frac{{{\mathrm{Odds}}\,\left( {pathogenic} \right)\,of\,a\,given\,critical\,domain\,or\,coldspot}}{{{\mathrm{Odds}}\,\left( {benign} \right)\,of\,a\,given\,critical\,domain\,or\,coldspot}},$$

given (Total variants + 1/2) cancel out

An estimated odds ratio of pathogenicity:

5 $$\frac{{({\mathrm{P}} + {\mathrm{LP}}) + 1/2}}{{({\mathrm{B}} + {\mathrm{LB}}) + 1/2}}$$

Confidence intervals were calculated using the log odds ratios method.^17^

Because most individuals clinically tested are affected, pathogenic variants are more likely to be represented in ClinVar. Thus, the estimated odds ratio of (P + LP)/(B + LB) will be skewed toward pathogenicity. However, for the purposes of coldspot identification, overestimating the frequency of pathogenic variants is conservative because this would make regions less likely to be classified as coldspots.

The (P + LP)/(B + LB) odds ratios for proposed coldspots were translated to suggested ACMG/AMP categories using a Bayesian framework, proposed in Tavtigian et al.^18^ with odds ratio of <0.48 considered “supporting benign,” <0.23 “moderate benign,” and <0.05 “strong benign.” Within this framework, proposed coldspots with odds ratios that translated to strong benign were considered confirmed coldspots.

A gene-wide Chi-squared test was performed to determine the expected distribution of VUS in coldspots compared with critical domains, given a chi-squared distribution. “Spacer regions” or areas between known critical domains and proposed coldspots were included for better coverage of the gene.

## RESULTS

### *BRCA1*

In *BRCA1*, 89.4% of missense variants submitted to ClinVar are classified as VUS and only 4.5% as pathogenic.^19^ A total of 89 pathogenic + likely pathogenic (P + LP), 119 benign + likely benign (B + LB), and 1759 VUS were included in the analysis, after excluding variants with CIP or no assertion criteria provided (Supplementary Data File).

**Fig. 1:**
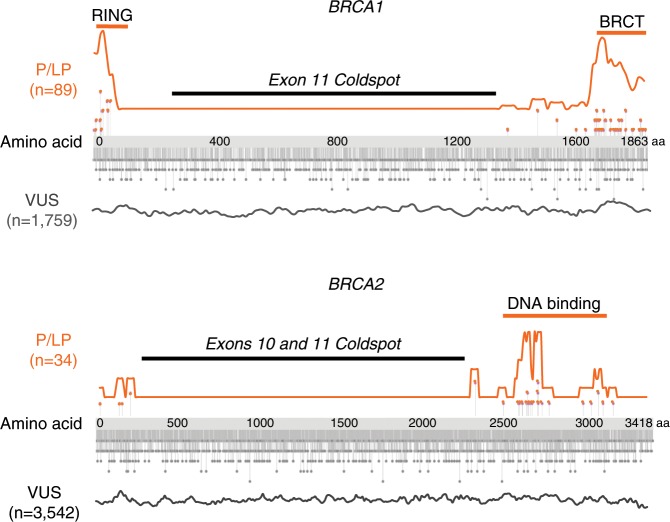
Distributions of missense variants in *BRCA1* and *BRCA2*. Missense variants are indicated by lollipops. For each gene, distributions of pathogenic and likely pathogenic (P/LP) missense variants are shown in orange above the gene and of variants of uncertain signficance (VUS) in gray below the gene. *BRCA1* exon 11 and *BRCA2* exons 10 and 11 harbor no P or LP missense variants and are defined as coldspots. We suggest reclassifying the 3115 VUS in these coldspots as likely benign.

Critical domains in *BRCA1* harbor the majority of P + LP missense variants, including the RING (29/89, 32.5%) and BRCT domains (53/89, 60.0%) (Fig. 1, top, and Table 1). The RING domain has 2.5% (3/119), and BRCT has 16% (19/119) of the B + LB variants. This corresponds to an estimated odds ratio of 8.4 (95% confidence interval 6.4–11.2) for the RING domain and 2.7 (95% confidence interval 0.9–8.6) for the BRCT domain. These odds of pathogenicity for missense variants correspond to “supporting pathogenic” according to Tavtigian’s Bayesian to ACMG/AMP category correlation (Table 2).^18^

By contrast, exon 11 accounts for ~60% of the coding sequence but has no confirmed P + LP missense variants, and 58.0% (69/119) of B + LB variants. The estimated odds ratio for pathogenicity of missense variants in exon 11 is <0.01 (95% confidence interval [CI] 0.0–0.01, which satisfies the criteria for “strong benign” evidence, Table 2).^18^ Therefore, we classify this region as a coldspot.

Despite a complete absence of pathogenic missense variants in exon 11, there is no evidence that coldspot information is currently being used for variant classification because missense VUS are reported about as commonly in exon 11 as in the critical domains (Table 1, Fig. 1). The 1048 VUS reported in the *BRCA1* exon 11 coldspot (Table 1) is about the same as the expected 1026 variants, assuming no difference in VUS rate compared with the critical functional domains. Restricting the analysis to variants with ≥2 star ClinVar ratings yielded similar results (Table S1).

All six putative P + LP missense variants in *BRCA1* that fall outside of critical domain regions are pathogenic because they impact splicing, not because of the amino acid change. *BRCA1* c.4484G>C (p.R1495T), c.4484G>A (p.R1495K), and c.4484G>T (p.R1495M) impact splicing at the exon 13 boundary; c.4675G>C (p.E1559Q) and c.4675G>A (p.E1559K) disrupt splicing at the exon 14 boundary; and c.4868C>G (p.A1623G) disrupts splicing in exon 16.^20–22^ Thus, 100% of P + LP missense variants either occur in a critical domain or affect splicing.

### *BRCA2*

For *BRCA2*, 94.4% of missense variants are classified as VUS and only 0.9% as P + LP.^19^ A total of 34 P + LP, 177 B + LB, and 3542 VUS missense variants were used in the analysis excluding variants with conflicting interpretations of pathogenicity (CIP) or no assertion criteria provided (Supplementary Data File). The majority of P + LP missense variants (25/34, 75%) fall within the highly conserved DNA-binding domain (Fig. 1, bottom).

*BRCA2* exons 10 and 11, which account for ~65% of the coding sequence, have no putative P + LP missense variants (0.0%), but contain 62.1% (110/177) of B + LB variation (odds ratio of <0.01, 95% CI 0.0–0.01, which satisfies the criteria for “strong benign” evidence, Table 2).^18^ Therefore we classify this region as a coldspot. As with *BRCA1*, restricting the analysis to variants with ≥2 star ClinVar ratings yielded similar results (Table S1).

Similar to *BRCA1*, the majority of *BRCA2* P + LP missense variants either occur in a critical functional domain or affect splicing. Known splice-disrupting variants include c.425G>T (p.S142I) and c.475G>A (p.V159M) at the last nucleotide of exons 4 and 5, respectively.^23,24^

The 2067 missense VUS reported in the *BRCA2* exon 10 and 11 coldspot (58.4% of total VUS) is about the same as the expected 2131 variants assuming there is no difference in VUS rate compared with the critical domains. Like *BRCA1*, coldspot reasoning is probably not being used for variant classification in *BRCA2* because the missense VUS rate is not meaningfully different in exons 10 and 11 compared with the critical domains (Table 1, Fig. 1).

## DISCUSSION

We suggest the term “coldspots” to describe regions of a gene that are tolerant of variation, where pathogenic missense variants are unlikely. We demonstrate that large coldspots exist in exon 11 of *BRCA1* and in exons 10 and 11 of *BRCA2*. Using a Bayesian framework where the odds of pathogenicity for each region are converted to ACMG/AMP^18^ classifications, we find that the missense patterns in *BRCA1* and *BRCA2* coldspots are consistent with “strong benign” evidence. This evidence could allow a new *BRCA1* exon 11 or *BRCA2* exon 10 or 11 missense variant to be initially classified as likely benign in most instances.

We acknowledge that there will be rare pathogenic variants in coldspots that are initially misclassified as likely benign by this approach, for example in regions that impact splicing. We suggest that the “coldspot” approach to initially classifying most variants as likely benign is akin to what is often done for deep intronic and intergenic regions of the genome. Some variants in these regions are pathogenic, but are rare enough to initially classify uncharacterized variants as likely benign. Just as with deep intronic regions, classification of missense variants in *BRCA1* and *BRCA2* coldspots should consider conservation of the variant position, the likelihood the variant could impact splicing, functional data, and clinical context.

Avoiding the initial VUS classification for many missense variants may prevent downstream negative consequences related to VUS for patients and physicians.^2^ A majority of breast cancer specialists reported feeling unsure about the clinical implications of a VUS, especially in the context of a negative family history.^9^ Among surgeons, 51% of lower-volume and 24% of higher-volume surgeons made the same treatment recommendation for a woman with a *BRCA1/2* VUS as a woman with a known pathogenic variant, and half of patients with a *BRCA1/2* VUS without a significant personal and/or family history of breast cancer underwent a bilateral prophylactic mastectomy.^10^ Moreover, VUS can increase patient anxiety and demand intensive counseling. Most patients interpret their *BRCA1/2* VUS as meaning there is some predisposition for cancer, despite recalling they were told the result is “noninformative.”^25^ Coldspot information could be used to avoid this problem by reducing the number of variants classified as VUS.

In existing guidelines, hotspots/critical domains are considered evidence to support pathogenicity (ACMG/AMP PM1), but coldspots are not used to support benign classification. We propose that an additional benign criteria for coldspots be included as part of future variant classification guidelines developed by ACMG/AMP. Currently, coldspot evidence could be used to support benign classification under the ACMG BP4 category in which multiple lines of computational evidence suggest no impact on the gene. Alternatively, odds ratios for coldspots could be used in quantitative multifactorial variant classification, as defined by Tavtigian or used by the ENIGMA consortium. We emphasize that our data support this approach only for *BRCA1* and *BRCA2* coldspots. Gene-specific expert panels, such as ClinGen,^26–28^ are well-suited to define the coldspot regions in additional clinically relevant genes, and to formally classify variants within proposed coldspot boundaries.

Ascertainment bias affects our analysis because missense variants in known critical domains are more likely to be followed up for definitive classification. This bias can be seen in *BRCA1*, as more functional studies exist for the RING domain,^29,30^ BRCT domain,^31,32^ and DBD domain^33–35^ compared with other regions of the gene.^14^ In addition, ClinVar data may not be representative of the population because variant reporting is voluntary. As variant data sharing improves,^36^ so will critical domain and coldspot characterization.^37,38^

Identification of coldspots could be assisted by reviewing the distribution of missense and synonymous variants in large population databases, by looking at evolutionary constraint. As anticipated, in *BRCA1* and *BRCA2* we find that the coldspot regions are less constrained than other regions of the genes. Using gnomAD population data, the ratio of missense to synonymous variants variants in the *BRCA1* and *BRCA2* coldspots is higher than other areas of the genes (*BRCA1*: 2.93 vs. 2.63; *BRCA2*: 3.32 vs. 2.96), indicating that the coldspot regions are under less constraint.

A generalized approach to identify coldspots within genes on a genome-wide basis is possible. Additional refinement of proposed coldspots can be made as more information and data become available with potential subdomain resolution for critical domains and coldspots. Alternative approaches that take into account the crystal structure and computation inferences of 3D conformation of protein to define spatial constraint^39^ may be informative in the future.

### Conclusion

We define coldspot regions in *BRCA1* and *BRCA2* in which most uncharacterized missense variants can be classified as likely benign rather than VUS. We propose that coldspot evidence be incorporated in variant interpretation guidelines to avoid systematic misclassification of these variants as VUS.

## URLs

**UCSC Genome Browser:** http://genome.ucsc.edu/

**DGV:** http://dgv.tcag.ca/dgv/app/home

**DECIPHER:** https://decipher.sanger.ac.uk/

**OMIM:** http://www.omim.org/

**ClinGen:** http://www.ncbi.nlm.nih.gov/projects/dbvar/clingen/

**ClinVar:** https://www.ncbi.nlm.nih.gov/clinvar/

**ExAC:** http://exac.broadinstitute.org/

**BRCA Exchange** http://brcaexchange.org

## Supplementary information

Supplementary Data File

Supplementary Online Materials

## Data Availability

The authors declare that all data supporting the findings of this study are available within the paper (and its supplementary information files).
