# Supplementary material for: Systematic misclassification of missense variants in BRCA1 and BRCA2 “coldspots”
Source: Genet Med. 2020 Jan 8;22(5):825–30. doi: 10.1038/s41436-019-0740-6 (PMC7200594; doi:10.1038/s41436-019-0740-6)
Supplement: Supplementary file 1 — Supplementary Data File [file 41436_2019_740_MOESM1_ESM.docx]

**SUPPLEMENTARY MATERIALS:**

**Supplementary Data File:** List of ClinVar variants and classifications used in analysis for *BRCA1* and *BRCA2*. There are two tabs in the workbook: “1. Used_in_analysis” lists only the variants used for the analysis, exluding 0-star variants that have no assertion criteria provided and variants with CIP; and “2. Full_includes_CIP_and_0_star” includes all missense variants. Note that *BRCA1* and *BRCA2* gene data is combined in both tabs.

**Table S1: ClinVar classification of missense variants in *BRCA1* and *BRCA2*, restricting to 2 star or greater rating.**

| Gene | Region | Codons | P or LP  N (%)^a^ | B or LB  N (%)^a^ | VUS  N (%)^a^ | Total  N (%)^a^ |  |
| --- | --- | --- | --- | --- | --- | --- | --- |
| *BRCA1* | Total missenses | 1-1863 | 67 (8.0) | 96 (11.4) | 678 (80.6) | 841 (100.0) |  |
|  | RING domain | 9-98 | 24 (38.1) | 3 (4.8) | 36 (57.1) | 63 (100.0) |  |
|  | BRCT repeats | 1649-1859 | 37 (28.2) | 15 (11.5) | 79 (60.3) | 131 (100.0) |  |
|  | Exon 11 | 224-1366 | 0 (0) | 54 (11.4) | 421 (88.6) | 475 (100.0) |  |
|  | Coiled-coiled | 1393-1424 | 0 (0) | 2 (14.3) | 12 (85.7) | 14 (100.0) |  |
| *BRCA2* | Total missenses | 1-3418 | 26 (1.6) | 123 (7.7) | 1456 (90.7) | 1605 (100.0) |  |
|  | Exon 10 and 11 | 226-2281 | 0 (0) | 84 (9.1) | 842 (90.9) | 926 (100.0) |  |
|  | BRC repeats | 1008-2082 | 0 (0) | 42 (8.1) | 475 (91.9) | 517 (100.0) |  |
|  | DNA binding | 2481-3186 | 19 (5.0) | 18 (4.7) | 345 (90.3) | 382 (100.0) |  |

Abbreviations: P or LP=pathogenic or likely pathogenic, B or LB=benign or likely benign, VUS=variant of uncertain significance. Transcripts are NM*_*007294.3 for *BRCA1* and NM_000059.3 for *BRCA2*. Variants were classified as described in the methods and last queried October 2019.

^a^Percent of P/LP, B/LB, and VUS in each region (by row), excluding variants with conflicting interpretations of pathogenicity.
